# Importance of controlling mesocotyl elongation in the development of rice seedlings intended for mechanical transplantation

**DOI:** 10.3389/fpls.2023.1213609

**Published:** 2023-10-04

**Authors:** Yuanli Jia, Tao Wang, Gaozi Zhou, Lei Tang, Xueping Yue, Xinrui Liu, Tao Cao, Juan Yang, Youfeng Tao, Fei Deng, Wei Zhou, Wanjun Ren, Yong Chen

**Affiliations:** Crop Ecophysiology and Cultivation Key Laboratory of Sichuan Province, Key Laboratory of Crop Ecophysiology and Farming System in Southwest China, Ministry of Agriculture, Sichuan Agricultural University, Chengdu, China

**Keywords:** mechanically transplanted rice, rice mesocotyl, seedling quality, root morphology, root coiling force

## Abstract

The mesocotyl facilities the emergence of deep-sown rice. However, the effects of mesocotyl elongation on mechanically transplanted rice seedlings remain unclear. In this study, the indica three-line hybrid rice Chuanyou 6709 (CY6709) and the indica conventional rice Guichao II (GCII) were selected as experimental materials. The seedlings were grouped based on mesocotyl lengths of 1.0 and 2.0 cm (M1 and M2, respectively), and seedlings without mesocotyl elongation were used as a control (M0). Seedling morphology, root morphology and physiology, and dynamic changes in soluble sugar and protein, malondialdehyde (MDA), and antioxidant enzyme activity in the mesocotyl were evaluated. The results showed that the elongation of mesocotyl is not conducive to improving the quality of mechanically transplanted seedlings, resulting in weak seedlings and poor root coiling force. The mesocotyl lengths of the seedlings showed a single peak with increasing seedling age, which gradually disappeared. The longer the mesocotyls, the slower their senescence. The MDA content of M2 was significantly lower than that of M1, and the activities of soluble sugar, soluble protein, and antioxidant enzymes of M2 were higher than those of M1, implying that seedlings with longer mesocotyls yielded lower-quality seedlings, reducing their suitability for mechanized transplantation. Compared with those of M0, the root-shoot ratio, stem base width, leaf age, leaf area, white root number, root coiling force, root length, root surface area, and root volume of M1 and M2 were reduced. Therefore, in the raising of rice seedlings, excessive elongation of the rice mesocotyl is not conducive to optimum root growth and development of aboveground structures for seedlings that are suitable for mechanized transplantation. Controlling the mesocotyl elongation can facilitate the cultivation of high-quality mechanically transplanted seedlings.

## 1 Introduction

Rice (*Oryza sativa L.*) is an important food crop worldwide (Krishnan et al., 2011). With the continuous development of agricultural mechanization in China, rice production has gradually transitioned from using traditional cultivation methods to implementing simple and efficient mechanized cultivation methods (Zhu and Chen, 2009). In the past 20 years, mechanized planting of rice in China, particularly mechanized transplanting, has developed rapidly. In 2019, mechanical transplantation was utilized in 94.54% of the total mechanically planted area (Li et al., 2018). Another important early stage of mechanized transplanting, rice transplanting technology, is also developing simultaneously. The cultivation of robust seedlings that are able to withstand mechanical transplanting is the basis for efficient mechanized seedling raising and transplanting (Yu et al., 2006). Mechanizing seedling raising can effectively improve quality and standardization, and reduce costs (Chen et al., 2020). The emergence of mechanized methods of raising seedlings in the dark results in a high seedling rate and robust seedlings (Chen et al., 2020). Under high-temperature, high-humidity, dark environments with disc pressure, seedlings can emerge quickly; however, long periods of darkness often result in excessive elongation of mesocotyls in rice seedlings (Li, 2016). The mesocotyl is the structure between the coleoptile node and seed base of the seedling. Mesocotyl elongation plays an important role in the processes of direct seeding, deep direct seeding, and deep seeding (Huang et al., 2010; Lee et al., 2012). The elongation of the mesocotyl pushes the seedling through the soil surface and may improve the anchorage and early vigor of the root (Hoshie et al., 2018). However, the effect of mesocotyl length on the quality of mechanically transplanted rice seedlings remains unclear. As a fibrous root crop, rice has three types of roots: the radicle (seed root), mesocotyl roots, and nodal roots (adventitious roots and crown roots). Mesocotyl roots are generally not present and occur only under deep sowing conditions or chemical treatment (Mao, 1986). Previous studies have focused on the relationship between mesocotyl elongation and seedling growth and development in direct rice seeding. The results showed that mesocotyl elongation length was positively correlated with internode length and plant height in directly seeded rice. Mesocotyl elongation can increase the stem wall thickness and support capacity of the plant base and enhance lodging resistance in the middle and late stages of plant growth. Moreover, with direct seeding, the root system has a deep distribution and wide area coverage and can efficiently use water (Jiang et al., 2017; Sun et al., 2020). However, in mechanized raising of rice seedlings, dark growing conditions lead to the occurrence of mesocotyl roots, which are not firmly rooted, thus affecting nutrient absorption, limiting dry matter accumulation and seedling growth (Wang et al., 2022), and further affecting the quality of seedlings for mechanical transplanting (Lin et al., 2016). However, there is a lack of relevant research on the impacts of mesocotyl elongation under the tray-overlaying method of raising rice seedlings within mechanized transplanting systems, and its effect on mesocotyl changes, root morphogenesis, seedling quality, and physiological characteristics is unclear. Therefore, in this study, rice seedlings were sown under the tray-overlaying method in two groups based on mesocotyl length, with a control group consisting of seedlings without mesocotyl elongation. This study aimed to examine the effects of rice mesocotyl elongation on the growth and development dynamics, seedling quality indices, soluble sugar, soluble protein, malondialdehyde (MDA), superoxide dismutase (SOD), peroxidase (POD), and catalase (CAT) contents, and other physiological indices of mechanically transplanted seedlings. The results of this study provide a theoretical basis for the regulation of mesocotyl elongation for more efficient seedling-raising.

## 2 Materials and methods

### 2.1 Materials

The tested rice varieties were Chuanyou 6709 (CY6709), a medium-grain, late-maturing, three-line hybrid indica rice, and Guichao 2 (GCII), a conventional indica rice. The CY6709 variety was provided by the Rice Research Institute of Sichuan Agricultural University and the GCII was provided by the Rice and Sorghum Research Institute of Sichuan Academy of Agricultural Sciences.

### 2.2 Methods

A single-factor completely randomized design was used in the experiment. Two groups were established with mesocotyl lengths of 1.0 and 2.0 cm, denoted as M1 and M2, respectively. Seedlings with no mesocotyl elongation (0 cm) were used as a control (M0). An indoor sand culture seedling test was carried out. First, 60 g of full and disease-free seeds were weighed. Then, the seeds were sterilized with a 5% sodium hypochlorite solution for 15 min, washed with ultrapure water three times, soaked for 24 h, and dried at room temperature until the grain surface was completely dry. Washed and dried river sand was placed in a plastic seedling hard disk (58 cm × 28 cm × 3.0 cm) with a bottom sand layer thickness of 1.8 cm. After the bottom sand was fully wetted with distilled water, the rice seeds were evenly sown and then covered with a 0.3 cm top layer of sand. After sowing, the seedling trays were stacked and placed in a dark 30°C temperature-controlled growth chamber for 5 days. After this period of dark growing conditions, the trays were placed in an outdoor open field for cultivation and fed a nutrient solution daily (Chen et al., 2019).

**Figure 1:**
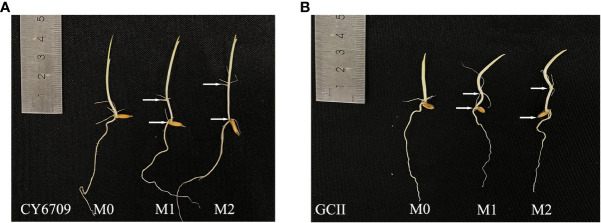
Mesocotyl length of **(A)** CY6709 and **(B)** GCII at a seedling age of 5 days. M0: no mesocotyl (control); M1: initial mesocotyl length, 1.0 cm; M2: initial mesocotyl length, 2.0 cm; white arrow = mesocotyl length.

Three groups of rice seedlings with different mesocotyl lengths were obtained by controlling the duration of darkness. After 5 days in darkness, seedlings with mesocotyl lengths of 0 cm (M0), 1.0 cm (M1), and 2.0 cm (M2) that exhibited uniform growth were artificially selected (**Figures 1A, B**). Each group consisted of 20 plants, with three replicates. A hydroponic system was used to allow observation of the growth dynamics of the rice mesocotyl. After selecting, the seedlings were wrapped with sponges at 1 cm above the mesocotyl and planted in a foam plastic plate with a pore size of 8 mm. The foam plastic plate was then placed in a nutrient solution-containing culture pot (40 cm × 30 cm × 12 cm), and the nutrient solution was replaced every 2 days. The nutrient solution was formulated according to the International Rice Research Institute (IRRI) formula (Chen et al., 2019). All seedlings were placed outdoors after planting, and the morphological changes of seedlings and mesocotyls were measured every 7 days. After each measurement, the seedlings of each treatment were carefully replanted in the culture basin to avoid artificial damage to seedlings during the planting process.

### 2.3 Sampling and measurements

#### 2.3.1 Seedling morphology

At days 5, 12, 19, 26, and 33 after sowing, 20 representative plants were selected to determine the seedling morphology, including seedling height, mesocotyl length, mesocotyl diameter, leaf area, leaf age, white root number of crown roots, white root number of mesocotyl roots, and stem base width, were measured. This process was performed in triplicate.

#### 2.3.2 Root-shoot ratio

At day 33 after sowing, 100 seedlings with the same growth vigor were selected for each group, and the plants were cleaned and cut to divide them into aboveground and root structures. After being placed in an oven at 105°C for 30 min, the samples were dried to a constant weight at 80°C. The dry weight of the aboveground structures and the roots of the 100 plants were measured, and the root-shoot ratio was calculated using the following equation:


(1) Root−shoot ratio = seedling root dry weight (g)/seedling shoot dry weight (g)

#### 2.3.3 Determination of root morphology

Five representative seedlings were selected at days 5, 12, 19, 26, and 33 after sowing and their root morphology were recorded. The total root length, total surface area, root tip number, root volume, and average root diameter of the seed roots and mesocotyl roots were scanned using an Epson V700 Photo scanner (Seiko Epson Corporation, Beijing, China) and analyzed with a WinRHIZO Root Analyzer System (Regent Instruments Inc, Quebec, Canada).

#### 2.3.4 Determination of root coiling force

At 33 day of sowing, three representative 10 cm × 10 cm seedling blocks were cut for each treatment, fixed at both ends, and one end was slowly hooked and pulled in the horizontal direction with an ELK-100 digital tensiometer. The reading displayed by the tensiometer when the seedling block broke was taken as the root coiling force (Lin et al., 2016).

#### 2.3.5 Mesocotyl physiological indices

The root activity was determined using the triphenyltetrazolium chloride method (Xiong, 2003). Representative plants were selected at 5, 12, 19, 26, and 33 days. After the roots of the plants were washed and dried, 0.2 g of the sample was weighed, and 10 mL of an equal amount of 0.4% triphenyltetrazolium chloride solution and 1/15mol/L phosphate buffer was added. The roots were fully immersed in the solution. The solution was kept at 37 °C for 2 h, and then 2 mL of 1 mol/L sulfuric acid was added to terminate the reaction. Finally, the root was taken out and wiped dry, and 10 mL ethyl acetate and a small amount of quartz sand were added to grind the sample in a mortar. The absorbance at 485 nm was measured and the root activity was determined.

The MDA content was determined using the thiobarbituric acid method (Chen et al., 2022). Representative plants were selected at 5, 12, 19, 26, and 33 days. Three portions of 0.2 g fresh mesocotyls were weighed in each treatment and placed in a mortar. 0.5% (w/v) thiobarbituric acid was added to grind the samples into a homogenate, and then heated in a boiling water bath for 10 min. After cooling, the homogenate was centrifuged at 3000 × *g* for 15 min. The absorbance values at 450, 532, and 600 nm were measured, and the MDA content was determined.

Soluble protein content was determined using the Coomassie Brilliant Blue G250 method (Zhang et al., 2022). Representative plants were selected at 5, 12, 19, 26, and 33 days. Three portions of 0.2 g fresh mesocotyls were weighed in each treatment and placed in a mortar, and 2 mL distilled water was added to grind the sample until homogeneous. The homogenate was centrifuged at 5000 r/min for 10 min, and then 0.1 mL supernatant, 0.9 mL distilled water and 5 mL Coomassie brilliant blue G-250 solution were mixed evenly with the sample. After standing for 2 min, the absorbance at 595 nm was measured and the soluble protein content was determined.

The soluble sugar content was determined using the anthrone method (Xiong, 2003). Representative plants were selected at 5, 12, 19, 26, and 33 days. Three portions of 0.2 g fresh mesocotyls were weighed for each treatment, and 5 mL distilled water was added. After immersion for 10 min in a boiling water bath, the samples were centrifuged at 3500 × *g* for 10 min. Then, 2 mL supernatant was taken and 5 mL anthrone sulfuric acid was added. After mixing, the absorbance value at 620 nm was measured and the soluble sugar content was determined.

The activity of SOD was determined using the nitrogen blue tetrazolium photochemical reduction method, and the absorbance at 560 nm wavelength was determined using an SOD kit (Beijing Solarbio Biotechnology) in ultraviolet spectrophotometer.

The POD activity was determined using the POD catalyzed H_2_O_2_ oxidation specific substrate method, and the absorbance at 470 nm wavelength was quickly determined using a POD kit (Beijing Solarbio Biotechnology) and analyzed using an ultraviolet spectrophotometer.

The CAT activity was determined using the CAT decomposition H_2_O_2_ method, and the absorbance at 240 nm wavelength was determined using an CAT kit (Beijing Solarbio Biotechnology) and analyzed using an ultraviolet spectrophotometer.

### 2.4 Statistical analysis

Microsoft Excel 2010 software was used for data collation, statistical software SPSS statistics v22.0 was used for data analysis and processing, least significant difference was used for multiple comparisons, and Origin 2021 was used for mapping.

## 3 Results

### 3.1 Morphological changes of mesocotyl during seedling growth

**Figure 2:**
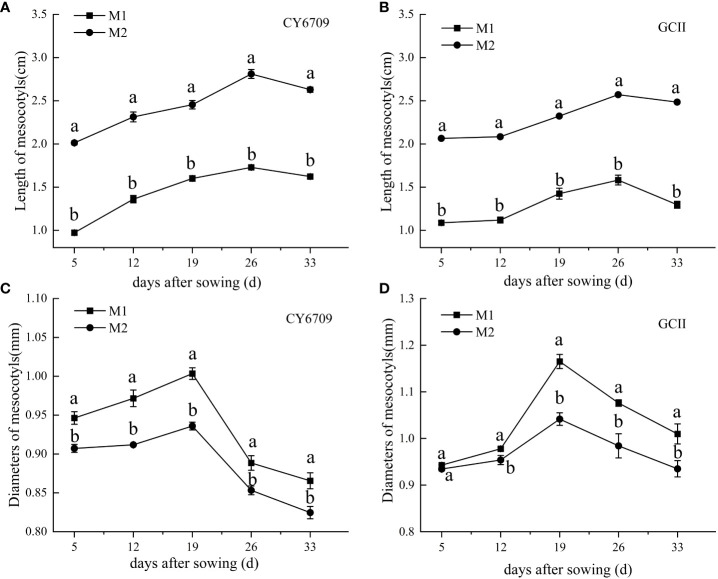
Morphological characteristics of seedling mesocotyls **(A)** mesocotyl length of CY6709; **(B)** mesocotyl length of GCII; **(C)** mesocotyl diameter of CY6709; **(D)** mesocotyl diameter of GCII. Each value represents the mean ± standard error. Each experiment was repeated three times. Different letters indicate significant difference among treatments at *P* < 0.05. Groups and abbreviations are the same as those given in **Figure 1**.

The mesocotyl length of the two rice varieties first increased and then decreased with increasing seedling age (**Figure 2**) and reached a maximum at a seedling age of 26 days, when the mesocotyl length of CY6709 in M1 and M2 was 1.73 and 2.81 cm, respectively (**Figure 2A**), and the mesocotyl length of GCII in M1 and M2 was 1.58 and 2.57 cm, respectively (**Figure 2B**). The mesocotyl diameter of CY6709 and GCII seedlings first increased and then decreased, reaching a maximum at a seedling age of 19 days. At 19 days, the mesocotyl diameters of CY6709 seedlings in M1 and M2 were 1.00 and 0.93 mm, respectively, (**Figure 2C**). The mesocotyl diameters of GCII seedlings in M1 and M2 were 1.17 and 1.04 mm, respectively, (**Figure 2D**).

### 3.2 Changes in mesocotyl physiological indices

#### 3.2.1 Changes in soluble sugar, soluble protein, and MDA content

**Figure 3:**
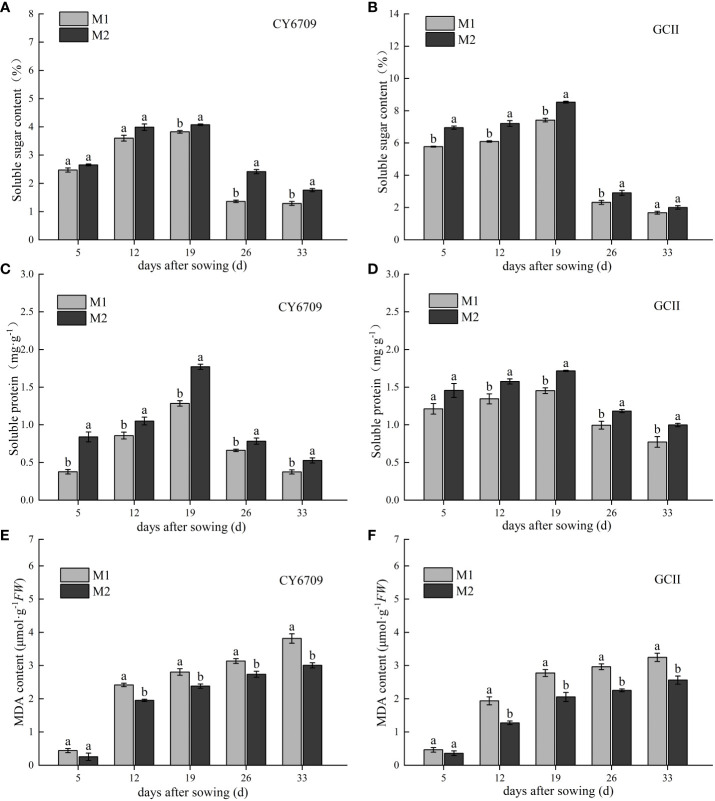
Changes in soluble sugar, soluble protein and malondialdehyde (MDA) during seedling mesocotyl senescence **(A)** changes in soluble sugar content of CY6709 groups; **(B)** changes in soluble sugar content of GCII groups; **(C)** changes in soluble protein content of CY6709 in different groups; **(D)** changes in soluble protein content of GCII groups; **(E)** changes in MDA content of CY6709 in different groups; **(F)** changes in MDA content of GCII groups. Each value represents the mean ± standard error. Each experiment was repeated three times. Different letters indicate significant difference among treatments at *P<* 0.05. Groups and abbreviations are the same as those given in **Figure 1**.

The soluble sugar and soluble protein content of CY6709 and GCII first increased and then decreased with the growth process (**Figure 3**), reaching a maximum at 19 days of seedling age, and then decreased rapidly. The soluble sugar and soluble protein contents in M1 were lower than those in M2. At 19 days, the contents of soluble sugar in M1 and M2 of CY6709 were 3.82% and 4.07%, respectively (**Figure 3A**), and the contents of soluble protein were 1.28·and 1.77 mg·g^-1^, respectively (**Figure 3C**). At 19 days, the soluble sugar contents in M1 and M2 of GCII were 7.42% and 8.53%, respectively (**Figure 3B**), and the soluble protein contents were 1.45 and 1.72 mg·g^-1^, respectively (**Figure 3D**). The dynamic change characteristics of the MDA content in the mesocotyls of the two rice varieties are shown in **Figures 3E, F**. The MDA content of mesocotyls in each group for the two varieties increased with increasing seedling age, and its accumulation at 5-12 days of seedling age was fast (**Figures 3E, F**). The results showed that, compared with M1, M2 showed slower aging, lower MDA content, and higher soluble sugar and soluble protein contents.

#### 3.2.2 Changes in the activity of the antioxidant enzyme system

**Figure 4:**
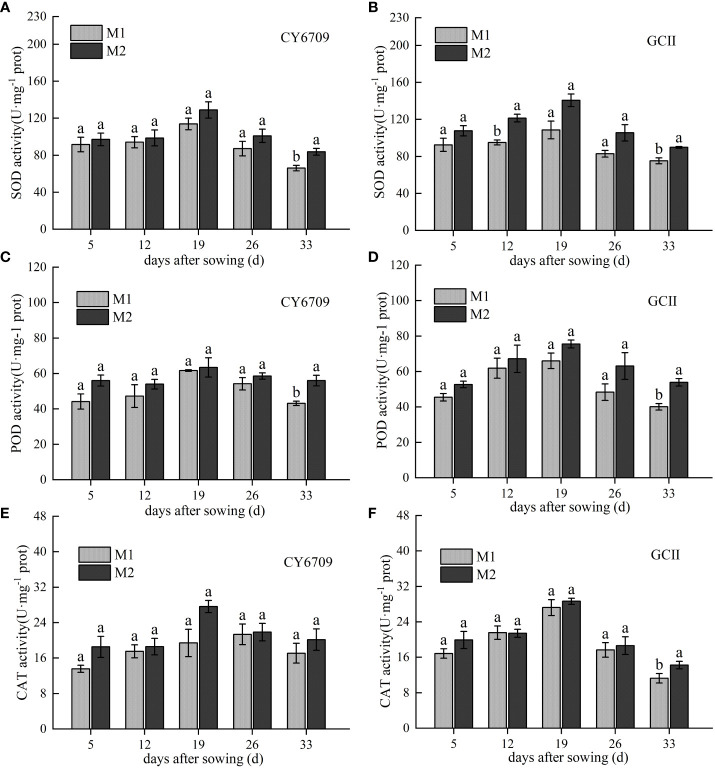
Changes in antioxidant enzyme activities in mesocotyls of different lengths **(A)** superoxide dismutase (SOD) activity of CY6709 mesocotyl; **(B)** SOD activity of GCII mesocotyl; **(C)** peroxidase (POD) activity of CY6709 mesocotyl; **(D)** POD activity of GCII mesocotyl; **(E)** catalase (CAT) activity of CY6709 mesocotyl; **(F)** CAT activity of GCII mesocotyl. Each value represents the mean ± standard error. Each experiment was repeated three times. Different letters indicate significant difference among treatments at *P*< 0.05. Groups and abbreviations are the same as those given in **Figure 1**.

The activities of SOD, POD, and CAT in CY6709 and GCII first increased and then decreased with increasing seedling age, peaking at 19 days (**Figure 4**). Compared with M1, M2 showed higher SOD, POD, and CAT activities. At a seedling age of 33 days, the activities of SOD and POD in M2 were significantly higher than those in M1 (**Figures 4A–D**). However, the CAT activity of GCII in the M2 group was significantly higher than that in the M1 group, and the CAT activity of CY6709 in the M2 group was higher than that in the M1 group; however, the difference was not significant (**Figures 4E, F**). These results indicate that, compared with M1, M2 exhibited higher antioxidant enzyme activity, higher ability to scavenge reactive oxygen species, and slower aging.

### 3.3 Dynamic changes in aboveground seedling growth

#### 3.3.1 Changes in plant height, leaf age, and leaf area

**Figure 5:**
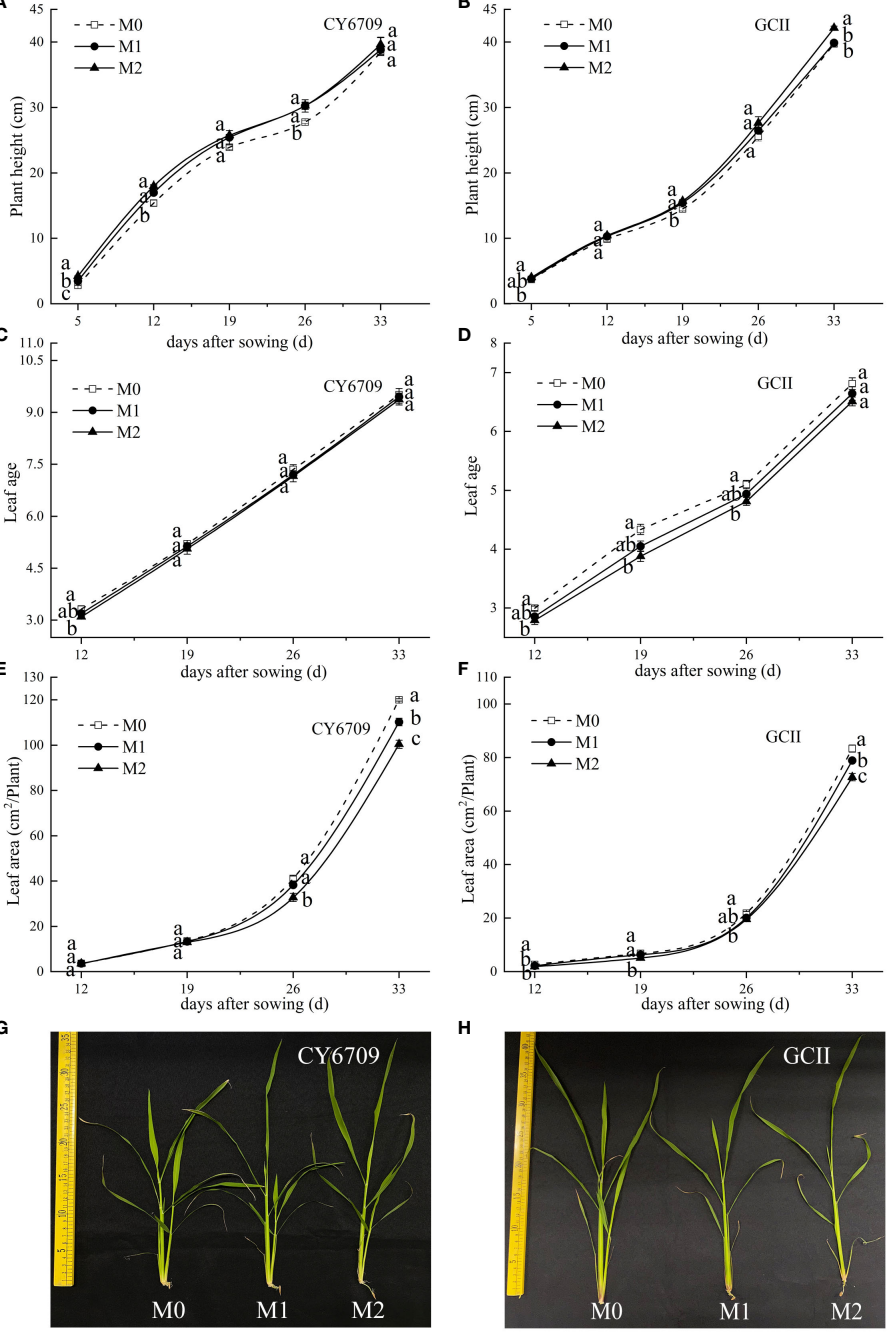
Effects of mesocotyls of different lengths on shoot growth dynamics of rice seedlings **(A)** plant height of CY6709 seedlings in each group; **(B)** plant height of seedlings in GCII groups; **(C)** leaf age of CY6709 seedlings in each group; **(D)** leaf age of seedlings in GCII groups; **(E)** leaf area of CY6709 seedlings in each group; **(F)** leaf area of seedlings in each group at GCII; **(G)** plant height of CY6709 seedlings in each group; **(H)** plant height of seedlings in GCII groups. Each value represents the mean ± standard error. Each experiment was repeated three times. Different letters indicate significant difference among treatments at *P<* 0.05. Groups and abbreviations are the same as those given in **Figure 1**.

Plant height, leaf age, and leaf area of the seedlings of the two varieties increased with mesocotyl growth (**Figure 5**). The plant height, leaf age, and leaf area of the mesocotyl grew slowly in the early stages of mesocotyl growth, and the growth of the mesocotyl accelerated in the late stages of senescence. The plant height of the two varieties, in descending order, was M2 > M1 > M0 (**Figures 5A, B**), whereas the leaf age and leaf area, in descending order, were M0 > M1 > M2 (**Figures 5C–F**). At 33 days, there was no significant difference in plant height between the CY6709 groups. The seedlings in the M2 group of GCII were significantly taller than those in M0 and M1, whereas there was no significant difference in leaf age between the groups of the two varieties. The leaf area of M0 was significantly larger than that of M1 and M2. Compared with M0, M2 showed greater plant height but smaller leaf age and leaf area, indicating that the longer the mesocotyl, the greater the plant height of the seedlings, and the smaller the leaf age and leaf area (**Figures 5G, H**).

#### 3.3.2 Changes of stem base width and thickness

**Figure 6:**
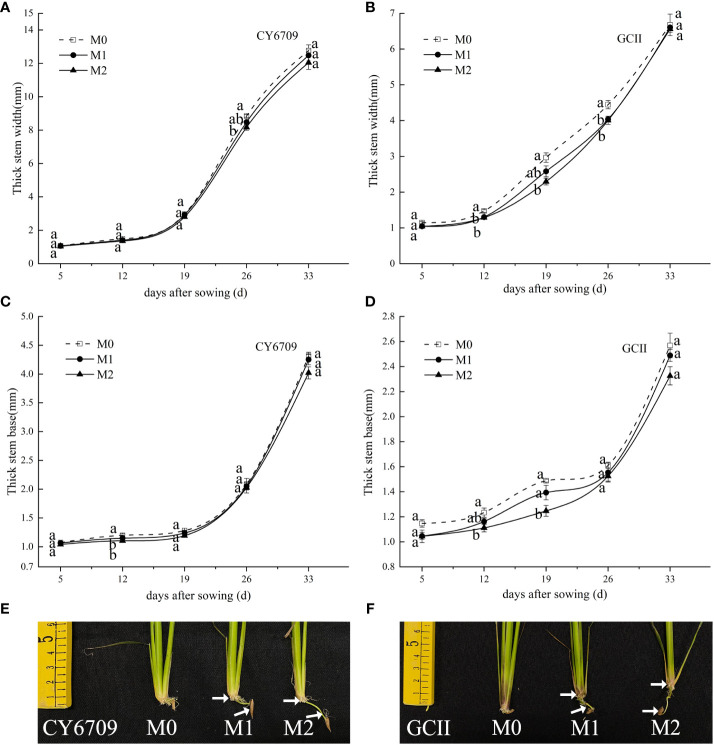
Effects of mesocotyls of different lengths on stem base width and stem base thickness of mechanically transplanted seedlings **(A)** stem base width of CY6709 seedlings in each group; **(B)** stem base width of GCII seedlings in each group; **(C)** stem base thickness of CY6709 seedlings in each group; **(D)** stem base thickness of GCII seedlings in each group; **(E)** stem base width of CY6709 seedlings in each group; **(F)** stem base width of GCII seedlings in each group. Each value represents the mean ± standard error. Each experiment was repeated three times. Different letters indicate significant difference among treatments at *P* < 0.05. Groups and abbreviations are the same as those given in **Figure 1**.

With the development of the rice mesocotyl, the stem base width and thickness of each group of the two rice varieties showed an increasing trend (**Figure 6**), and the growth of stem base width and thickness was accelerated in the late stage of mesocotyl senescence. The width and thickness of both varieties, in descending order, were M0 > M1 > M2 among seedling groups with different mesocotyl lengths (**Figures 6A–D**). Compared with M0, M2 exhibited smaller stem base width and thickness, indicating that a longer mesocotyl was not conducive to improving the stem base quality of rice seedlings. The longer the mesocotyl, the thinner the stems of the rice seedlings (**Figures 6E, F**).

### 3.4 Effects of different mesocotyl lengths on root growth dynamics of seedlings

#### 3.4.1 Changes of white root number, root activity, and MDA content

**Figure 7:**
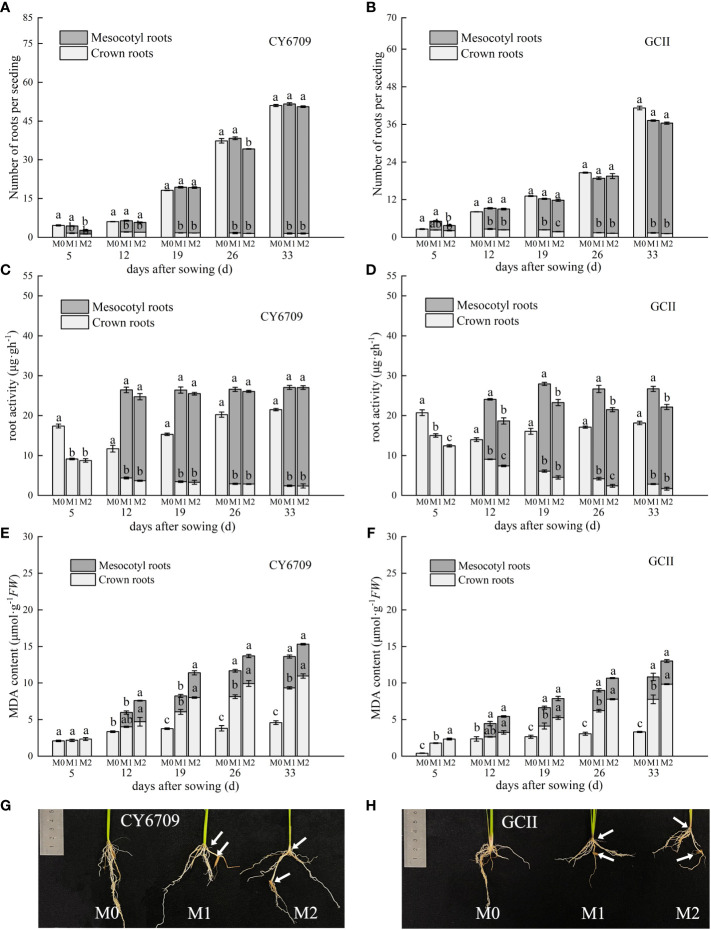
Effects of different lengths of mesocotyls on rice roots **(A)** white root number of CY6709 seedlings; **(B)** white root number of GCII seedlings; **(C)** root activity of CY6709 seedlings; **(D)** root activity of GCII seedlings; **(E)** malondialdehyde (MDA) content in roots of CY6709 seedlings; **(F)** MDA content in roots of GCII seedlings; **(G)** root phenotype of CY6709 seedlings at 33 days seedling age; **(H)** root phenotype of GCII seedlings at 33 days seedling age. Each value represents the mean ± standard error. Each experiment was repeated three times. Different letters indicate significant difference among treatments at *P*< 0.05. Treatments and abbreviations are the same as those given in **Figure 1**.

Changes in white root number, root activity, and root MDA content of seedlings with different mesocotyl lengths are shown in **Figure 7**. The results show that the total number of white roots of the two varieties showed an increasing trend, and the number of white roots of the mesocotyl roots increased with increasing seedling age, with a greater number in M1 than in M2. The white root number of crown roots showed an increasing trend with increasing seedling age in M0, whereas those in M1 and M2 showed a downward trend, with a greater number in M1 than in M2. The number of mesocotyl white roots in both test varieties increased with increasing seedling age. The number of white roots in M1 and M2 showed a downward trend, but the number of white roots in M0 showed an increasing trend (**Figures 7A, B**).

Root activity is an important index for evaluating seedling health. As shown in **Figures 7C, D**, the root activity of the mesocotyl roots of the two test varieties increased with increasing seedling age, and the mesocotyl root activity of M1 was greater than that of M2, which in turn was lower than that of M0. The root activity of the crown roots decreased with increasing seedling age, and the crown root activity of M1 was greater than that of M2, which in turn was lower than that of M0. At 33 days, the root activity of M0 was significantly higher than that of M1 and M2, but there was no significant difference between that of M1 and M2. Furthermore, there was no significant difference in the mesocotyl root activity between M1 and M2.

The results of the dynamic changes in MDA content in the roots showed that the content of the two varieties increased with increasing seedling age, and the content of the two varieties in M0 was lower than that in M1 and M2. The MDA content of the crown roots in M1 and M2 was higher than that of the mesocotyl roots (**Figures 7E, F**). At day 33, the MDA content of the crown roots of the two varieties of M2 was significantly higher than those of M1 and M0. There was no significant difference in the MDA content of mesocotyl roots between M1 and M2, but the contents of both were lower than that of M0. As the seedlings grew, the number of white roots in the crown roots of seedlings with elongated mesocotyls decreased, root activity decreased, accumulation of MDA increased, roots gradually aged, and number of mesocotyl roots increased. The longer the mesocotyl, the lower the number of crown and mesocotyl roots, resulting in seedlings with root morphogenetic traits that were not optimal for mechanical transplantation (**Figures 7G, H**).

#### 3.4.2 Changes of root morphological characteristics

**Figure 8:**
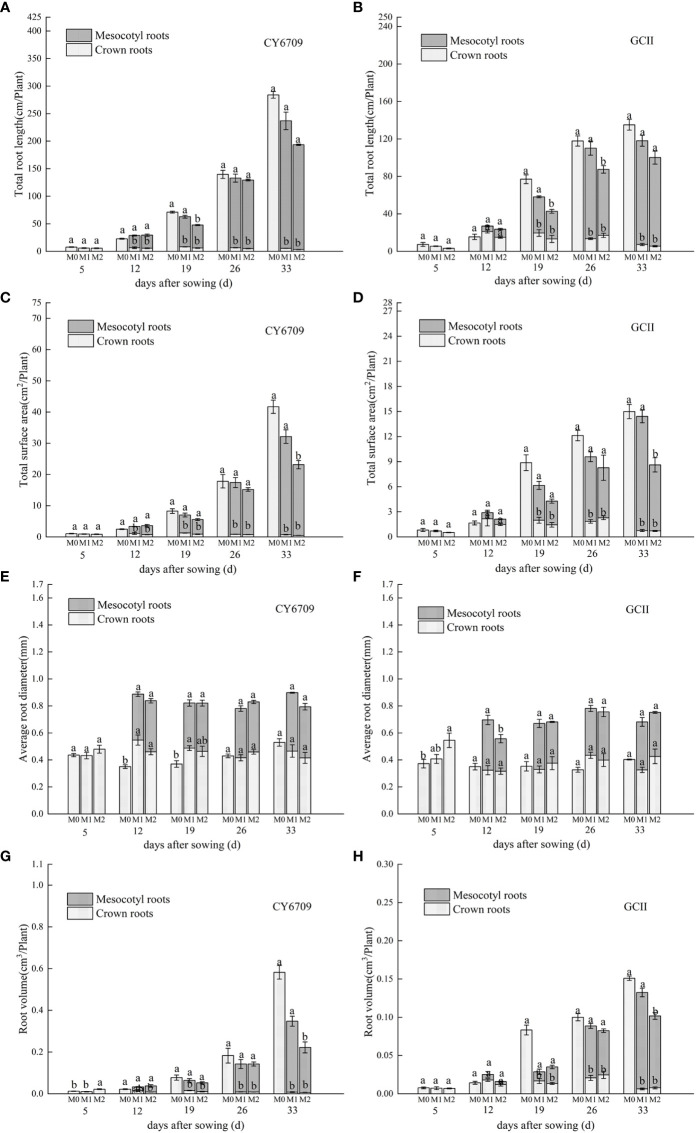
Effects of mesocotyls of different lengths on root morphology of machine-transplanted seedlings. **(A)** total root length of CY6709 seedlings in each group; **(B)** total root length of seedlings in GCII groups; **(C)** total surface area of CY6709 seedlings in each group; **(D)** total surface area of seedlings in GCII groups; **(E)** average root diameter of CY6709 seedlings in each group; **(F)** average root diameter of seedlings in each group at GCII; **(G)** root volume of CY6709 seedlings in each group; **(H)** root volume of seedlings in GCII groups. Each value represents the mean ± standard error. Each experiment was repeated three times. Different letters indicate significant difference among treatments at *P*< 0.05. Groups and abbreviations are the same as those given in **Figure 1**.

The characteristics of root morphological changes in seedlings with mesocotyls of different lengths are shown in **Figure 8**. The results show that the two varieties had the same trends. With increasing seedling age, the total root length, root surface area, and root volume of M0 increased continuously. The total root length, root surface area, and root volume of the mesocotyl roots of M1 and M2 increased continuously with increasing seedling age, whereas the same characteristics in crown roots first increased and then decreased. The total root length, root surface area, and root volume in the M1 group was higher than that in the M2 group. Therefore, seedlings with longer mesocotyls had limited root growth and reduced total root length, root surface area, and root volume.

#### 3.4.3 Changes in root-shoot ratio and root coiling force

**Figure 9:**
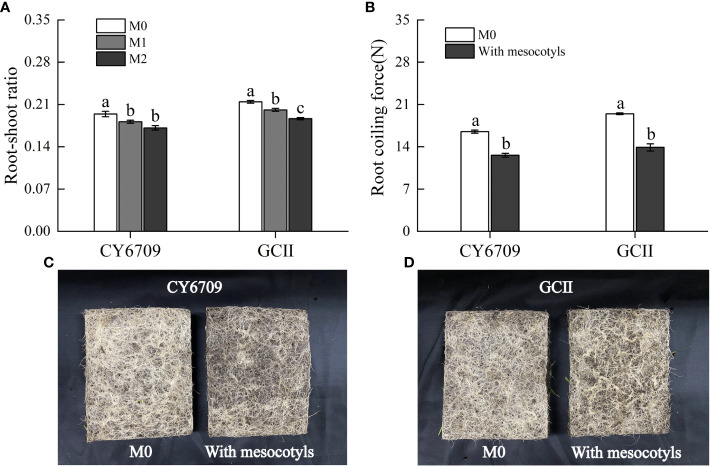
Effect of mesocotyl elongation on root-shoot ratio and root coiling force of mechanically transplanted seedlings **(A)** root-shoot ratio of CY6709 and GCII groups; **(B)** root coiling force of CY6709 and GCII groups; **(C)** root coiling force of CY6709 at 33 days seedling age; **(D)** root coiling force of GCII at 33 days seedling age. Each value represents the mean ± standard error. Each experiment was repeated three times. Different letters indicate significant difference among treatments at *P<* 0.05. Groups and abbreviations are the same as those given in **Figure 1**.

The longer the mesocotyl, the smaller the root-shoot ratio and root coiling force of the two varieties of seedlings (**Figure 9**). At day 33, the root-shoot ratio of seedlings with mesocotyls of different lengths, in descending order, was M0 > M1 > M2, and the performance among varieties was consistent (**Figure 9A**). The root-shoot ratio of M0 was significantly higher than those of M1 and M2. There was no significant difference in the root-shoot ratio between the M1 and M2 groups of CY6709; however, in GCII, the root-shoot ratio in M1 was significantly higher than that in M2. In mechanized seedling production, the seedlings contain both long mesocotyl seedlings and short mesocotyl seedlings, and the root coiling forces of seedlings with and without mesocotyl elongation were measured. The results of root coiling force evaluation showed that the root coiling force of seedlings with mesocotyl elongation was significantly lower than that of seedlings without mesocotyl elongation (**Figure 9B**), and the performance was consistent among the two varieties. Therefore, mesocotyl elongation in seedlings affected root growth, which in turn reduced the root coiling force (**Figures 9C, D**); thus affected the root quality of seedlings suitable for mechanical transplanting.

#### 3.4.4 Effect of mesocotyl on seedling growth

**Figure 10:**
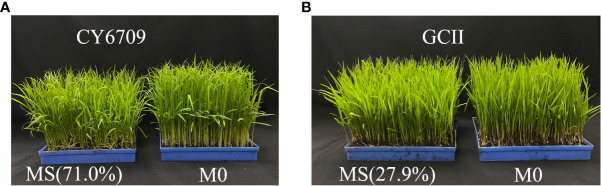
Effect of mesocotyl elongation on rice seedling growth **(A)** growth of CY6709 seedlings at 33 days of seedling age; **(B)** growth of GCII seedlings at 33 days of seedling age MS: With mesocotyl seedlings (M1+M2), M0: no mesocotyl seedlings (control).

The effect of mesocotyl elongation on the growth of rice seedlings is shown in **Figure 10**. The proportion of seedlings with mesocotyls in the seedling tray to the total seedlings (the number of seedlings without mesocotyls + the number of seedlings with mesocotyls) was measured. It was found that 71.0% of the CY6709 seedlings had mesocotyls, while 27.9% of the GCII seedlings had mesocotyls. The shoot growth of MS (M1+M2) was observably poorer than that of M0. This demonstrated that the shoot growth of seedlings with elongated mesocotyls was slower and the seedlings were weak.

## 4 Discussion

### 4.1 Effect of mesocotyl elongation on seedling morphogenesis

Studies have shown that rice seeds grow mesocotyls and produce mesocotyl roots under deep sowing conditions or in response to chemical treatments. Mesocotyl roots begin to appear approximately 7d after sowing, and some roots appear after the mesocotyls are fully elongated (Shigeo and Eizo, 1978). The fibrous root system of rice is an important organ for absorbing nutrients and water, and a metabolic organ for material exchange with the aboveground parts (Li et al., 2022). The growth and development of the root system directly affect aboveground traits and yield (Zhao et al., 2023). Previous studies have shown that, under the stress of deep sowing, rice seeds may adjust their energy distribution through a physiological response mechanism to reduce the growth of roots, thereby ensuring the elongation of germs in order for them to be unearthed as soon as possible. However, deep sowing has a negative impact on seedling growth in the form of root dysplasia, which manifests as short primary root length, small root-shoot ratio, reduced root weight, and light root weight, posing a great threat to seedling quality (Wang, 2007). Desirable root traits at the seedling stage are the basic means for forming strong seedlings. This study demonstrated that seedlings with long mesocotyls exhibited inhibited root morphogenesis and physiological characteristics. With increasing seedling age, the number of white roots, root activity, total root length, root surface area, and root volume gradually decreased (**Figures 7G, H**); additionally, compared with those of M0, the root-shoot ratio and root coiling force of M1 and M2 seedlings of the two varieties decreased (**Figures 9C, D**). Elongation of the mesocotyl affects the coordinated growth of the aboveground and underground plant parts. The longer the mesocotyl, the greater the degree of disruption to the aboveground growth and the greater the reduction of the underground growth, which is manifested in a longer embryo and a shorter primary root (Wang, 2007) (**Figures 1A, B**). The results of this experiment showed that, with an increase in seedling age, the seedling height showed a trend of M2 > M1 > M0, and the seedling mesocotyl length in all groups showed a trend of an initial increase followed by a decrease, reaching a maximum at 26 days. However, before day 26, the difference in plant height between M0, M1, and M2 of CY6709 was great, and the difference in plant height became smaller after that, indicating that the mesocotyl had started to age and had an effect on the plant height of the seedlings (**Figures 5G, H**). In addition, the elongation of the mesocotyl also inhibited the leaf growth of seedlings to a certain extent. After mesocotyl senescence began at 26 days, the leaf age and leaf area of M0 increased rapidly compared to those of M1 and M2. In short, the root development of seedlings with long mesocotyls was poor, which directly affected the shoot growth of seedlings, resulting in poor seedling quality and weak seedlings (**Figures 6E, F**).

### 4.2 Physiological characteristics of mesocotyl senescence

After plant senescence, a variety of reactive oxygen species are produced in plants, which leads to oxidative stress. The balance between the generation and scavenging of free radicals in cells is disrupted, which eventually leads to aggravation of membrane lipid peroxidation (Quirino et al., 2000; Guo et al., 2007; Ren et al., 2023). SOD, CAT, and POD are important protective enzymes of the active oxygen scavenging system, which can effectively prevent the increase in active oxygen, prevent membrane lipid peroxidation, and delay plant senescence (Zhen et al., 2019). Previous studies have shown changes in active oxygen metabolism during the senescence of adzuki bean leaves. The results showed that the activities of SOD, POD, and CAT decreased with leaf senescence, whereas the MDA content increased with leaf senescence (Song et al., 2010). This study showed that the mesocotyl reached its longest length at 26 days of seedling age, and then began to senesce rapidly. The MDA content in the mesocotyl increased with increasing seedling age, and the activities of SOD, POD, and CAT first increased and then decreased. At 33 days, the activities of SOD, POD, and CAT in mesocotyls decreased, while the content of MDA increased. This is mainly because the decrease of enzyme activity caused the disorder of active oxygen metabolism in the membrane system, an increased electrolyte leakage rate, and an increase in the superoxide anion (O^2-^) and H_2_O_2_ production rates, resulting in the increase of membrane lipid peroxidation products in the mesocotyl, the inhibition of mesocotyl growth, and the acceleration of mesocotyl senescence (Song et al., 2010; Zafar et al., 2021). Soluble sugar and soluble proteins are nutrients in plants, and the nutrient content in plants changes greatly during senescence (Zhang, 2019). As a primary source of fuel for cell metabolism, carbohydrates provide energy for other types of metabolism and provide energy for maintaining the balance of the intracellular environment.

The soluble sugar content is used as an indicator of the activity level of cell metabolism (Riyaz et al., 2014). Previous studies have shown a significant positive correlation between the elongation length of the mesocotyl and soluble sugar content (Ma et al., 2014). This study showed that the soluble sugar content in the mesocotyl of the M2 group was higher than that of M1, indicating that the longer the mesocotyl, the more vigorous its material metabolism. Most soluble proteins in plants are metabolic enzymes, which are involved in various metabolic processes in plants, and their levels in plants represents the level of metabolic activity. (Zhang, 2019). This study showed that the longer the mesocotyl, the higher the soluble protein content. The MDA content of M2 was lower than that of M1, and the activities of antioxidant enzymes and nutrient content of M2 were higher than those of M1. The seedlings with long mesocotyls exhibited slower hypocotyl senescence than did those with short mesocotyls, which was due to the higher accumulation of harmful MDA substances in short mesocotyls, a lower activity of protective enzymes, and decreased nutrients. In summary, the reason for the deterioration in seedling quality of mechanically transplanted seedlings with long mesocotyls is that the slower the senescence of long mesocotyls, the slower the transportation of nutrients and water absorbed by roots to the aboveground parts. Simultaneously, excessive elongation of the mesocotyl implies that the upper part of the mesocotyl is exposed to the soil surface, which is not conducive to the development of mesocotyl roots, resulting in slower growth of the aboveground parts and weaker seedlings. The seedling groups with short mesocotyls used the mesocotyl roots to absorb nutrients and water in the soil after the mesocotyls disappeared, which was beneficial to the growth and development of the aboveground parts of the seedlings (**Figures 10A, B**).

### 4.3 Necessity of regulation of mesocotyl elongation to improve the quality of seedlings for mechanical transplantation

High seedling quality improves the overall quality of mechanical transplanting processes by shaping high-quality field populations and ensuring high-quality and efficient production of mechanically transplanted rice (Yu et al., 2006). Healthy seedlings are also key to the high quality and high yield of machine-transplanted rice (Zhang, 2020). It is generally believed that strong seedlings for mechanical transplanting require a seedling height of 12–18 cm, seedling age of 15–25 days, leaf age of 2.8–4.0 days, thick stem base, high degree of plumpness, high root-shoot ratio, strong root activity, good coiling, and certain seedling age elasticity (Du, 2008). Previous studies have shown that seedlings that are too tall affect the quality of mechanical transplanting (Lin et al., 2016). When the seedlings are too tall, the transplanter cannot catch the root when catching the seedlings, resulting in seedlings getting folded, which will lower the quality of mechanical transplanting in the field (Zhang et al., 2008). The weakness of seedlings can also affect the quality of mechanical transplantation and prolong the regreening time of seedlings after entering the field environment (Hu et al., 2013). Root coiling force is also a key indicator affecting the quality of machine transplanting. If the root coiling force is too small, the seedling may break easily, which can affect the seedling growth speed and transplanting quality (Yu et al., 2006). This study showed that, compared with seedlings without mesocotyls, the longer the mesocotyl, the taller the seedling; however, the stem base width and stem base thickness were smaller, the seedling width was thinner, and the seedling qualities were therefore less desirable for mechanical transplantation. In addition, the total root length, root surface area, and total root volume of long mesocotyl seedlings were decreased, root activity was not high, and the root-shoot ratio decreased, when compared to those in M0. The root systems of long mesocotyl seedlings exhibited poor carpeting, small root coiling force, and easy dispersal of seedlings, which are undesirable qualities for seedlings intended for roll handling and mechanical transplanting. Moreover, the ability of seedlings to resist plant injury during mechanical transplanting was poor, and the number of roots after transplanting was small. Root activity was weak and the root development of seedlings after transplanting was slow, which is not conducive to effective nutrition and water absorption, resulting in slow seedling regreening and slow seedling emergence. Therefore, regulating mesocotyl elongation is an important means of cultivating robust seedlings suitable for mechanical transplanting and improving the quality of mechanical transplanting during the production process of mechanized seedling emergence and raising.

## 5 Conclusions

The mesocotyl of rice showed a trend of senescence with increasing seedling age, and the physiological indices of rice mesocotyls varied with mesocotyl length. We found that, with an increase in the degree of mesocotyl elongation, the MDA content was reduced, while the soluble sugar and protein contents and antioxidant enzyme activities increased accordingly, indicating that the longer the mesocotyl, the slower the senescence. In addition, a longer mesocotyl leads to uncoordinated growth and development of the aboveground and underground parts of rice seedlings. The seedlings were weak, the root morphology and root coiling ability were poor, and the seedlings lacked the robust qualities that are desirable for seedlings intended for mechanical transplantation. Therefore, controlling the elongation of dark-grown rice mesocotyls is necessary to improve the quality of mechanically transplanted seedlings.

## Data availability statement

The original contributions presented in the study are included in the article/supplementary material. Further inquiries can be directed to the corresponding author.

## Author contributions

YJ: Data curation, Investigation, Methodology, Writing –original draft, Writing – review & editing. TW: Data curation, Investigation, Methodology, Supervision. GZ: Data curation, Investigation. LT: Data curation, Investigation. XY: Data curation, Investigation. XL: Data curation, Investigation. TC: Data curation, Investigation. JY: Data curation, Investigation. YT: Software. FD: Software. WZ: Software. WR: Methodology, Supervision. YC: Conceptualization, Supervision, Writing – review & editing. All authors contributed to the article and approved the submitted version.

## References

[B1] Chen C. Gong H. Q. Jin M. C. Gao J. H. (2019). Correlation between root morphology and accumulation of phosphorus in rice seedlings under different N forms. Chin. J. Rice Sci. 33, 167–175. doi: 10.16819/j.1001-7216.2019.8059

[B2] Chen H. Z. Xiang J. Wang Y. J. Xu Y. C. Chen Y. P. Zhang Y. K.. (2020). Seedlings emergence characteristics and transplanting quality using the Tray-Overlaying seedlings raising mode in rice mechanized transplanting systems. J. Nucl. Agric. Sci. 34, 2823–2830. doi: 10.11869/j.issn.100-8551.2020.12.2823

[B3] Chen X. Y. Chen Y. J. Zhang W. Zhang W. L. Wang H. Zhou Q. P. (2022). Response characteristics of root to moisture change at seedling stage of *Kengyilia hirsuta* . Front. Plant Sci. 13. doi: 10.3389/fpls.2022.1052791 PMC9853184 36684787

[B4] Du H. P. (2008). Rice mechanized production technology manual (Shanghai: Shanghai Science and Technology Publishers).

[B5] Guo F. Wan S. B. Wang C. B. Wu Z. F. Zhen Y. P. (2007). Nitrogenase and protective enzyme activities during the yield-stage of high-yield peanut intercropped with wheat. Acta Botanica Boreali-Occidentalia Sinica 27, 309–314.

[B6] Hoshie O. Niño P. M. C. B. Crisanta S. B. Jun-ichi K. Taiken N. Aurora M. C.. (2018). Longer mesocotyl contributes to quick seedling establishment, improved root anchorage, and early vigor of deep-sown rice. Field Crops Res. 228, 84–92. doi: 10.1016/j.fcr.2018.08.015

[B7] Hu R. Wang J. J. Qin Y. B. Wang Z. M. AOYang Y. N. (2013). Effect of soilless substrate seedling raising machine transplanting on continuous cropping late rice. China Rice 19, 103–105.

[B8] Huang C. Jiang S. K. Feng L. L. Xu Z. J. Chen W. F. (2010). QTL analysis for mesocotyl length in rice (Oryza sativa L.). Acta Agronomica Sinica 36, 1108–1113.

[B9] Jiang J. F. Jin L. C. Wang L. R. (2017). Research progress in elongation of mesocotyl in rice. Acta Agriculturae Jiangxi 29, 23–26. doi: 10.19386/j.cnki.jxnyxb.2017.12.05

[B10] Krishnan P. Ramakrishnan B. Reddy K. ,. R. Reddy V. R. (2011). High-temperature effects on rice growth, yield, and grain quality. Adv. agronomy 111, 87–206. doi: 10.1016/B978-0-12-387689-8.00004-7

[B11] Lee H. S. Kang J. W. Chung N. J. Ahn S. N. (2012). Identification of molecular markers for mesocotyl elongation in weedy rice. Kor J. Breed Sci. .44, 238–244.

[B12] Li Y. N. (2016). Study on influences of light and temperature and bulked segregation analysis for mesocotyl elongation in rice (Shanghai: Shanghai Ocean University).

[B13] Li G. Fu P. Cheng G. Lu W. Lu D. (2022). Delaying application time of slow-release fertilizer increases soil rhizosphere nitrogen content, root activity, and grain yield of spring maize. Crop J. 10, 1798–1806. doi: 10.1016/j.cj.2022.04.014

[B14] Li Z. H. Ma X. Li X. H. Chen L. T. Li H. W. Yuan Z. C. (2018). Research progress of rice transplanting mechanization. Trans. Chin. Soc. Agric. Machinery 49, 1–20. doi: 10.6041/j.issn.1000-1298.2018.05.001

[B15] Lin Y. J. Zhang J. H. Hu J. J. Zhu L. F. Cao X. C. Yu S. M.. (2016). Effects of different seedling substrates on physiological characters and grain yield of mechanized-transplanted rice. Trans. Chin. Soc. Agric. Engineering 32, 18–26. doi: 10.11975/j.issn.1002-6819.2016.08.003

[B16] Ma D. R. Kong D. X. Liu X. L. Gao Q. Ding G. H. Zhao M. H.. (2014). Mesocotyl elongation of weedy rice and its relationship with grain amylase activities and soluble sugar contents. Chin. J. Rice Sci. 28, 97–102.

[B17] Mao L. Z. (1986). Adaptation of rice to flooded soil. Bull. Biol. 6, 8–10.

[B18] Quirino B. F. Noh Y. S. Himelblau E. Amasino R. M. (2000). Molecular aspects of leaf senescence. Trends Plant Sci. 7, 278–282. doi: 10.1016/s1360-1385(00)01655-1 10871899

[B19] Ren B. Yu W. Liu P. Zhao B. Zhang J. (2023). Responses of photosynthetic characteristics and leaf senescence in summer maize to simultaneous stresses of waterlogging and shading. Crop J. 11, 269–277. doi: 10.1016/j.cj.2022.06.003

[B20] Riyaz A. D. Inayatullah T. Syed S. A. (2014). Sugars and sugar alcohols have their say in the regulation of flower senescence in Dianthus chinensis L. Sci. Hortic-Amsterdam 174, 24–28. doi: 10.1016/j.scienta.2014.04.003

[B21] Shigeo N. Eizo M. (1978). On the formation of adventitious roots and callus tissues in rice mesocotyl. Japanese J. Crop Sci. 47, 163–164. doi: 10.1626/jcs.47.163

[B22] Song H. Feng B. L. Gao X. L. Gao J. F. Wang P. K. Chai Y.. (2010). Leaf senescence and reactive Oxygen metabolism in different Adzuki Bean Cultivars (Lines). Acta Agronomica Sinica 36, 347–353. doi: 10.3724/sp.j.1006.2010.00347

[B23] Sun P. Wei X. Y. Cai H. W. (2020). QTL mapping of the first internode length in rice at seedling stage under dark condition. Mol. Plant Breeding 18, 6755–6761. doi: 10.13271/j.mpb.018.006755

[B24] Wang N. (2007). Study on mesocotyl elongation characteristics of weedy rice in Northeast China (Shenyang: Shenyang Agricultural University).

[B25] Wang Y. L. Li H. Lan T. M. Zhu D. F. Chen H. Z. Wu H. (2022). Effect of covering soil thickness in seedling tray on the rice seedlings growth for machine transplanting. Acta Agriculturae Universitatis Jiangxiensis 44, 311–318. doi: 10.13836/j.jjau.2022032

[B26] Xiong Q. E. (2003). Plant Physiology Experiment Tutorial (Chengdu: Sichuan Science and Technology Publishers).

[B27] Yu L. H. Ding Y. F. Xue Y. F. Ling Q. H. Yuan Z. H. (2006). Factors affecting rice seedling quality of mechanical transplanting rice. Trans. Chin. Soc. Agric. Engineering 22, 73–78. doi: 10.3321/j.issn:1002-6819.2006.03.016

[B28] Zafar S. A. Uzair M. Khan M. R. Patil S. B. Fang J. Zhao J. F.. (2021). *Dps1* regulates cuticle development and leaf senescence in rice. Food Energy Secur. 10, e273. doi: 10.1002/fes3.273

[B29] Zhang J. W. (2019). Changes of physiological and biochemical indexes in flower organs of hemerocallis during senescence (Shanxi: Agricultural University).

[B30] Zhang Z. (2020). The effect of exogenous regulator on the seedling quality of rice associated with long-age seedings of machine transplanting (Nanjing: Agricultural University).

[B31] Zhang S. Q. Guo X. L. Li J. Y. Zhang Y. H. Yang Y. M. Zheng W. G.. (2022). Effects of light-emitting diode spectral combinations on growth and quality of pea sprouts under long photoperiod. Front. Plant Sci. 13. doi: 10.3389/fpls.2022.978462 PMC9490185 36161035

[B32] Zhang Z. J. Wang J. Lang Y. Z. Yu L. H. Xue Y. F. Zhu Q. S. (2008). Growing characteristics of rice seedlings of over-optimum age for mechanical transplanting. Acta Agronomica Sinica 34, 297–304. doi: 10.3724/SP.J.1006.2008.00297

[B33] Zhao J. Lai H. Bi C. Zhao M. Liu Y. Li X. D.. (2023). Effects of paclobutrazol application on plant architecture, lodging resistance, photosynthetic characteristics, and peanut yield at different single-seed precise sowing densities. Crop J. .11, 301–310. doi: 10.1016/j.cj.2022.05.012

[B34] Zhen X. Y. Yang J. Q. Li X. X. Liu Z. X. Gao F. Zhao J. H.. (2019). Effects and physiological mechanisms of sowing depth on the growth progress and leaf senescence of peanut. Acta Agronomica Sinica 45, 1386–1397. doi: 10.3724/sp.j.1006.2019.94074

[B35] Zhu D. F. Chen H. Z. (2009). Rice transplanting development and food security. China Rice 15, 4–7. doi: 10.3969/j.issn.1006-8082.2009.06.002

